# Specialty of Dentistry

**Published:** 1887-09

**Authors:** 


### The Specialty of Dentistry.

An Address by Prof. A. W. Smith before the Kentucky State Dental Association.

*Mr. President and Gentlemen:*

The honor of welcoming you to-day has been delegated to me, and my position upon this occasion reminds me very forcibly of the following anecdote ;

During the administration of Mr. Buchanan the daughter of a prominent senator was married at Washington. The wedding was a grand affair, attended by the President, members of the Cabinet, and other distinguished people. When the ceremony was concluded Mr. Buchanan stepped forward to offer his congratulatians and imprinted a sounding kiss upon the lips of the bride. A member of Congress standing near-by inquired, "Mr. President is it understood that I am to follow suit?" "No sir," answered Mr. Buchanan "it is understood that I kiss for the Nation." On *this* occasion, therefore, it is understood that I speak for my colleagues, and on their behalf bid you hearty welcome to our hearts and homes.

It is a proud moment indeed when we look around us and see the distinguished guests whose presence honors our halls to-day: members of an association which has done so much to advance the standard of the specialty of Dentistry in this and other States, and who assemble here now, as if to show their approval of our young institution, and encourage us to persevere in the work we have undertaken. Gentlemen *again* I bid you welcome, but words seem cold and lifeless when the heart is deeply stirred, and our gratification on seeing you among us cannot be adequately expressed in language.

In making this address of welcome I am at a loss *what* to speak about in order to interest you most. When I make this statement, however, I know you will not retort like the man, who, when an orator exclaimed "Gentlemen, what *shall* I speak about?" answered "Mr. speak about five minutes." Ah, well judging you by myself, I am confident there is *no* subject better calculated to interest you than the one which is nearest to our hearts, namely, the Specialty of Dentistry.

Not six months ago we threw open the doors of the Hospital Dental College to those students who desired to become dentists in the highest, and truest sense of the word. No glittering bribes were held out—no promise of speedy graduation after an insufficient course of study—but we *did* promise to do our duty by our pupils and dismiss them *only* after a searching examination had proved them competent to pursue their calling. It has been, and will be our object to promote the progress of the Dental Specialty, and so elevate the standard of our profession that the dentist of the future shall be an ornament and honor to his calling.

The Dental Associations of the United States have been vastly instrumental in encouraging and forwarding this noble object. Too much cannot be said in praise of their labors, and, as year follows year, and victory succeeds victory, the world at large will more fully appreciate the benefits conferred by these admirable associations.

If the member of any profession will look back to other times, and contrast the present with the past, he cannot but realize what immense strides have been made, what great changes effected since the pioneers of his profession first practiced and preached in the worldly wilderness. He will also notice that only the honorable, true, conscientious practitioners have been proven pure gold in the crucible of time. The sluggard, the quack, the unambitious mechanic have arisen and flourished in their day, but that day was soon ended, and remembrance has stricken their names off her scroll as unworthy to survive. O how truly, how justly, has Emerson exclaimed:

> " What is excellent,
> As God lives is permanent."

We can best judge of the progress made by a nation when we examine the professional, social, and business life of its people, then, *when* we find the representatives of any calling beneficial to mankind, honored and respected, it is almost infallibly a sign of true advancement. It indicates clearer insight into the human character, broader way of thinking, and a nobler estimate of mankind. We realize also, that all these beneficial changes were effected by the unselfish efforts of sincere men, professionals who so *loved* their calling, so labored, so struggled against prejudice and ignorance that the barriers raised by bigotry were insensibly broken down, and the heroes of an honest cause at last recognized and rewarded. Sometimes, alas, rewards came too late to gladden the hearts of the men who suffered and hoped to obtain them, but they *never* came too late to encourage the other workers for the same cause and therefore were not wasted. Such men as these did not pause to ask, "What good will my work do me?" but, "What good will my work do my profession?" This idea they kept constantly in view, and their ambition was all the higher, the nobler, because it was so unselfish.

The standard of a profession is raised or debased by the character and work of its representatives. It rests entirely with them whether they will assert and maintain their claims to their true position or allow the public to estimate them as it pleases. Therefore, in making choice of a profession this question should be solemnly asked of our conscience, "Am I fitted for this work? Will it be of as much use to my fellow-beings as to myself?" A great many, however, question themselves in an entirely different way, and ask, "Will this profession bring me in more money than any other?" If the answer be in the affirmative they push ahead, and with only a mercenary idea in view entirely disregard their probable unfitness for the duties so rashly undertaken.

These men are the traitors in the camp of any profession, more dangerous, more subtle, more destructive than any avowed enemy.

Some professions were originally the outcome of man's necessities, others of his love of luxury or amusement; but as "the times change and we change with them," so likewise does the standard of a profession alter, but *generally* for the better. Take the drama, for instance; there was a time when an actor was a creature for men to scorn. Laberius, a Roman knight, committed suicide when forced by Cæsar to appear upon the stage. Shakespeare in one of his sonnets deplores his humiliating profession of a player. Moliere was equally sensitive on the same subject. Even at this day some people still entertain a prejudice against the wearers of "the sock and buskin." The majority, however, point with pride to their dramatic and lyric stars, fete them, entertain them, and envy them that power which moves men to smiles or tears at will. Medical and surgical specialists were not always honored as they are now. There have been times when they who pursued these callings occupied an inferior social position, until mental advancement and the efforts of enlightened men and women brought about an improved state of affairs, and to-day practitioners of every specialty of medicine can hold up their heads with the proudest and call the highest in the land their equals.

The dental specialty, practiced originally by the most famous physicians of early times, has been wrongfully degraded from its high estate and dragged through the mire by sordid quacks and superficial operators, who, utterly ignoring the higher requirements of dentistry, thereby lowered their professional standing and social status.

Better times, however, are dawning, better men coming to the front, better thinkers and workers are banding themselves together, resolved to convince the world at large that dentistry is a distinct branch of medical science, and must be recognized and honored as such.

In America especially this improved state of affairs is noticeable. As beacon after beacon springs up amid the darkness, until the bright flame upon one hill is answered by another far away, even so have schools and associations sprung into existence all over the country, and the darkness of ignorance has been illumined by their steady radiance. The mighty ægis of the law now shields and protects them, while national approbation honors them as national necessities.

The American dentist is making a name for himself everywhere. The most exclusive, the most lucrative practice in Europe has been built up by an American, whose work has proved his superiority over other professionals. It is this honest skilled work, coupled with a thorough knowledge of medicine and surgery, which makes the true dentist of to-day, and, let us hope, will make the dentist of the future. Such dentists, gentlemen, it is our aim, our ambition, to send out from this collage to benefit their fellow-creatures. We desire that this institution may be a lode-star to the members of the profession, a rallying place for the association of Kentucky and her sister States, and its doors will stand open to admit them at all times and all seasons.

Gentlemen, when a noted divine was asked, "how long should a sermon last," he answered, "not longer than fifteen minutes, with a leaning to the side of mercy." I suppose the same rule may be observed in regard to an address, so I will bring mine to a conclusion.

Once more I bid you welcome to our beautiful city, the pride, the ornament of a glorious State. May your brief sojourn amongst us be as pleasant to you as it is to us. The "old Kentucky homes," so famed in song and story, throw open their doors, leave the latch-string hanging outside, while warm hearts and outstretched hands bid you welcome in the name of hospitality.

